# Absorption of iron from edible house crickets: a randomized crossover stable-isotope study in humans

**DOI:** 10.1093/ajcn/nqac223

**Published:** 2022-08-26

**Authors:** Martin N Mwangi, Dennis G A B Oonincx, Marijke Hummel, Dessy A Utami, Lidyawati Gunawan, Margot Veenenbos, Christophe Zeder, Colin I Cercamondi, Michael B Zimmermann, Joop J A van Loon, Marcel Dicke, Alida Melse-Boonstra

**Affiliations:** Division of Human Nutrition and Health, Wageningen University & Research, Wageningen, Netherlands; Training Research Unit of Excellence, Blantyre, Malawi; Laboratory of Entomology, Wageningen University & Research, Wageningen, Netherlands; Animal Nutrition Group, Wageningen University & Research, Wageningen, Netherlands; Division of Human Nutrition and Health, Wageningen University & Research, Wageningen, Netherlands; Global Alliance for Improved Nutrition, Utrecht, Netherlands; Division of Human Nutrition and Health, Wageningen University & Research, Wageningen, Netherlands; Faculty of Health Science, Esa Unggul University, Jakarta, Indonesia; Division of Human Nutrition and Health, Wageningen University & Research, Wageningen, Netherlands; Nutriolab, Jakarta, Indonesia; Laboratory of Entomology, Wageningen University & Research, Wageningen, Netherlands; CLM Research and Advice, Culemborg, Netherlands; Human Nutrition Laboratory, Institute of Food, Nutrition and Health, Swiss Federal Institute of Technology in Zürich (ETH Zürich), Zürich, Switzerland; Human Nutrition Laboratory, Institute of Food, Nutrition and Health, Swiss Federal Institute of Technology in Zürich (ETH Zürich), Zürich, Switzerland; Wyeth Nutrition Science Centre, Nestlé Nutrition Institute, Vevey, Switzerland; Human Nutrition Laboratory, Institute of Food, Nutrition and Health, Swiss Federal Institute of Technology in Zürich (ETH Zürich), Zürich, Switzerland; Laboratory of Entomology, Wageningen University & Research, Wageningen, Netherlands; Laboratory of Entomology, Wageningen University & Research, Wageningen, Netherlands; Division of Human Nutrition and Health, Wageningen University & Research, Wageningen, Netherlands

**Keywords:** iron, edible insects, *Acheta domestica*, stable isotopes, fractional iron absorption, bioavailability, chitin, anemia, human, Netherlands

## Abstract

**Background:**

Edible insects are a novel source of animal protein. Moreover, edible insects contain iron concentrations similar to meat, potentially making them a valuable iron source for human consumers. Yet, it is unknown to what extent iron from insects is absorbed in humans.

**Objectives:**

In this exploratory study, we assessed fractional iron absorption from house crickets (*Acheta domesticus*) consumed with refined (low-phytate, noninhibiting) or nonrefined (high-phytate, inhibiting) meals.

**Methods:**

Intrinsically [^57^Fe]-labeled and control crickets were reared. Six iron-balanced experimental meals were randomly administered crossover to 20 iron-depleted females (serum ferritin <25 µg/L; 18–30 y old), in 2 time-blocks of 3 consecutive days, 2 wk apart. Three meals consisted of refined maize flour porridge with either [^57^Fe]-labeled crickets, [^58^Fe]SO_4_ (reference meal), or unlabeled crickets plus [^54^Fe]SO_4_. The other 3 meals consisted of nonrefined maize flour porridge with the same respective additions. Blood samples were drawn to assess the 14-d isotope enrichment in erythrocytes, and meal-specific fractional iron absorption was calculated. In vitro digestion was used to explore possible explanations for unexpected findings.

**Results:**

Mean fractional iron absorption from ^57^Fe-labeled house crickets with refined maize porridge (3.06%) and from refined maize porridge with unlabeled crickets (4.92%) was lower than from the reference meal (14.2%), with respective mean differences of −11.1% (95% CI: −12.6%, −9.68%) and −9.29% (95% CI: −10.8%, −7.77%). Iron absorption from all meals based on unrefined maize porridge was low (<3%), and did not differ for the 2 meals with crickets compared with the reference meal. In vitro digestion showed that chitin, chitosan, and calcium limited iron bioaccessibility to a large extent.

**Conclusions:**

Iron absorption from house crickets and fortified maize porridge with crickets is low, which may be explained by the presence of chitin and other inhibitors in the cricket biomass.

This trial was registered at https://www.trialregister.nl as NL6821.

## Introduction

The global prevalence of anemia is high, with approximately one-third of all women of reproductive age being affected. Anemia is mostly the result of insufficient intake of absorbable dietary iron (1). Heme iron in the form of hemoglobin and myoglobin (found in meat, fish, poultry, and dairy products) is well absorbed (∼25%), whereas nonheme iron (mostly found in plant-based food) is poorly absorbed (<10%) (2). When consumed within the same meal, ascorbic acid and muscle tissue are enhancers of nonheme iron absorption, whereas phytate, polyphenols, calcium, and milk protein act as inhibitors (2). In low-income societies, animal foods tend to be unaffordable, and most calories are derived from grains and legumes containing high amounts of phytate and other inhibitors (3, 4).

In its report on how to feed the world by the year 2050, the FAO calls for entomophagy (eating of edible insects) as a novel approach to diversify the human diet (5). Consumption of edible insects has the potential to sustainably improve intake of essential nutrients because of their high protein and mineral content, and because their production is less harmful to the environment than that of meat (6–12). Although >2000 insect species are consumed by humans (13, 14), their potential contribution to human iron intake and absorption has not yet been investigated systematically. The house cricket (*Acheta domesticus* L.) is a popular species commonly consumed in Asia and Africa (14, 15), and contains more iron per 100 g than beef, pork, and chicken (16, 17). In insects, the primary form of heme iron is present in small amounts of cytochromes with a presumed bioavailability similar to iron from myoglobin and hemoglobin (18), whereas nonheme iron is mostly ferritin-bound and is abundantly present, with unknown bioavailability to humans. An in vitro study reported the bioaccessibility of iron from house crickets to be comparable with sirloin beef (19). Like vertebrate meat, protein from insects may enhance nonheme iron absorption [known as the “meat factor”; (2)], although several compounds may also act as antinutritional factors that can chelate and bind free minerals and prevent their uptake in the gut.

In this exploratory study, we aimed to investigate the fractional absorption of iron from intrinsically iron-labeled house crickets consumed with an inhibitory or noninhibitory meal, and to compare this with iron absorption from reference meals without crickets. We also aimed to assess iron absorption from extrinsically labeled meals with (unlabeled) house crickets added. We hypothesized that iron absorption from meals with house crickets would be superior.

## Methods

### Production of house crickets (*Acheta domesticus* L.; Orthoptera: Gryllidae)

Approximately half of the house crickets for this study were produced at the Laboratory of Entomology (Wageningen University), and the other half were supplied by a commercial cricket producer (Nostimos) following similar procedures. Both batches were pooled to a total of 6.7 kg unlabeled and 12.9 kg ^57^Fe-labeled crickets. Crickets were reared in a climate chamber at 30°C, with a dark/light regime of 12 h/12 h and relative air humidity of 50%. Cricket eggs, laid in small containers with moistened vermiculite, were collected daily and transferred into a large plastic box until hatching (55 × 38 × 28 cm, BAUHAUS Twente). Half carton egg trays were placed vertically in the box to increase surface area. A water dispenser (Gebroeders de Boon) provided the crickets with drinking water, and the dispenser opening was filled with a paper tissue to prevent crickets from drowning. Crickets received slices of carrot daily (mainly as a source of liquid) and a basal chicken feed diet (Kuikenopfokmeel 1, Kasper Faunafood) ad libitum during the first 2 wk. Subsequently, they received an experimental diet (Research Diet Services) with added unlabeled Fe or isotopically labeled ^57^Fe until they reached the adult stage. Water and feed were checked daily and replenished when necessary to provide an ad libitum supply. One day after the first adult cricket was seen in a box (after 31–34 d), crickets were relocated into a new box. They received only carrots for 24 h, to remove the experimental diet from their gut. After 24 h, crickets were harvested and stored frozen at −21°C.

For the labeled cricket diet, ^57^Fe-metal (96.7% enrichment) was purchased from Chemgas. A total of 2.9942 g ^57^Fe-metal was added to 5892 μL sulfuric acid (96% concentrated) and 59 mL milli-Q water. The mixture was left under argon at room temperature until the iron had dissolved. This solution was sprayed at 1 bar with an H1/4VV-650067 nozzle over 2 kg experimental cricket feed (Research Diet Services) in a vacuum coater (Type 305, Dinnissen Process Technology) at the feed processing facility of Wageningen University. This mixture was then dried overnight at 50°C. The 2-kg mixture containing the ^57^FeSO_4_ was then added to 48 kg unlabeled feed (Research Diet Services) and mechanically mixed for 5 min (Type F60, Halvor-Forberg). A similar procedure was followed for the control diet, except that, instead of isotopic iron, 13.940 g unlabeled FeSO_4_ heptahydrate (Sigma-Aldrich) was added and hand-mixed with 5 kg of the basal diet. This mixture was then mechanically mixed with 45 kg of the basal diet for 5 min. Both experimental diets were then packed in paper bags, sealed with plastic tape, and stored at −18°C until use.

### Study participants

We recruited Dutch females aged 18–30 y through a volunteer database kept at Wageningen University & Research, Netherlands. Prospective participants (*n* = 139) received a verbal explanation of the study during information sessions. They were also invited to taste a nonlabeled maize porridge enriched with ground house crickets so that they could get familiar with the taste, texture, and aroma of the study meals. Each volunteer received an explanation of the study in writing, and willing volunteers were invited for a screening visit after signing an informed consent form (*n* = 106). To be eligible for participation in this study, participants were required to *1*) be of marginal iron status (serum ferritin concentration <25 µg/L), because iron depletion increases the capacity to absorb iron, hence it amplifies the effect size; *2*) have a C-reactive protein (CRP) concentration <5.0 mg/L; *3*) not be severely anemic (hemoglobin concentration ≥80 g/L); *4*) have a body weight ≤65 kg (this was to prevent substantial variance in blood volume and thereby in the amount of circulating iron between participants, to make sure that even low absorption would be detectable); *5*) not be allergic to edible insects, crustaceans, or mites; *6*) not be pregnant or lactating; *7*) not be experiencing metabolic, chronic, or hematologic diseases; *8*) not have undergone a blood transfusion, blood donation, or experienced severe blood loss over the past 6 mo; *9*) not be taking long-term medication except for contraceptives; and *10*) not consume vitamin and mineral supplements within 2 wk before the study and be willing not to do so until the end of the study. This study was registered in the Netherlands Trial Register (https://www.trialregister.nl/trial/6821), Trial ID: NL6821, source ID: NL59400.081.16. The Medical Research Ethics Committee of Wageningen University (METC-WU), Netherlands, approved the study, and all study procedures were conducted in accordance with the Helsinki Declaration of 1975 and its later revisions.

### Study design and procedures

The study was conducted in February–March 2018 in the research facility of the Division of Human Nutrition and Health, Wageningen University, Netherlands. Participants came to the research facility for a total of 9 visits comprising the initial information and tasting session, screening, study meal administration (6 visits), and endpoint visit. Standard methods were used to measure body weight and height. Refined (low-phytate) and unrefined (high-phytate) maize porridge was used as the food vehicle because it represents a staple food commonly consumed in Sub-Saharan Africa; thus, if bioavailability would prove to be good, house crickets could be used to enrich such commonly consumed porridge diets to improve iron status. Each participant received 6 different experimental meals provided in 2 series (1st series: days 7–9; 2nd series: days 23–25). Because of the volume, experimental meals were divided into 2 portions of 400–450 g each, which were served at breakfast time (07:00–09:00) and ≥3 h after the first serving (10:00–12:00). Two hours after consuming the second portion of the study meal, participants were given a lunch meal with restricted quantities of carbohydrates, fats, and proteins to facilitate normal digestion. To optimize iron absorption from the meals, participants were neither allowed to consume anything between the first and second portions of the meal nor between the second portion and the provided standardized lunch. Participants were provided with 500 mL bottled water (Spa Reine, Spadel Nederland BV) and ad libitum salt, pepper, and noncaloric artificial sweetener with each meal. Meals were prepared a day before administration and reheated in a microwave (800 W) for 1 min and 10 s. Meal weight, including bowl and spoon, was recorded before and after being served to participants to determine the exact amount consumed. Duplicate meals were collected twice for all meal types and stored frozen (−20°C) for later analysis.

#### Randomized meal allocation

We conducted a single-blind, randomized, partial Williams design crossover isotope study with 6 experimental meals, where each participant acted as their own control. Because only 3 stable-isotope labels, i.e., ^54^Fe, ^57^Fe, and ^58^Fe, were available to label the 6 experimental meals, the Williams crossover design was partial to allow use of the same isotope twice for different test meals, and meals with the same isotope could not be administered to a participant during the same feeding series. Therefore, 290 meal administration schemes were possible for this study. Participants were randomly allocated to 1 of these unique meal administration schemes by randomly generated numbers in Microsoft Excel (2016; Microsoft Corporation).

#### Blood collection

Fasting venous blood samples were collected from each participant at screening (day 0), midpoint (day 23), and at the last study day (day 39). Blood samples were collected using a 3-mL K_2_EDTA tube (Vacutainer, Becton-Dickinson), a 3-mL Lithium Heparin tube (Vacutainer, Becton-Dickinson SST^TM^), a 3.5-mL uncoated serum separator tube (Vacutainer, Becton-Dickinson), and a 3-mL false bottom tube (INPECO) to assess concentrations of hemoglobin, serum ferritin, soluble transferrin receptor (sTfR), C-reactive protein (CRP), and erythrocyte iron isotopes.

#### Experimental meals

Experimental meals were composed of maize meal porridge with or without crickets. Maize porridge was prepared from either refined (30 mg phytate/100 g) or nonrefined (600 mg phytate/100 g) maize flour (6.75% flour, 93.25% water), which resulted in an average phytate:Fe molar ratio of 0.12 and 2.2 in the refined and nonrefined maize porridges, respectively. Edible house crickets (*Acheta domesticus*), intrinsically labeled with ^57^Fe, were added to 1 pair of meals. Similarly, nonlabeled house crickets were added to another pair of meals and labeled extrinsically with ^54^Fe (as FeSO_4_). For comparison, we also prepared a pair of refined and nonrefined maize porridge meals without crickets but enriched with ^58^Fe (as FeSO_4_). The total iron content in each meal was matched by adding unlabeled FeSO_4_ (**Table 1**).

**TABLE 1:** Ingredients, isotope labels, and iron content of the experimental meals

	Meals with refined maize flour^1^			Meals with nonrefined maize flour^2^		
	[^57^Fe]-crickets	No crickets	Unlabeled crickets	[^57^Fe]-crickets	No crickets	Unlabeled crickets
Maize porridge						
Weight, g	800	800	800	800	800	800
Ground crickets						
[^57^Fe]-labeled, g	100	0	0	100	0	0
Nonlabeled, g	0	0	50	0	0	50
Fe content^3^						
From crickets						
Fe, mg	7.51	0	3.89	7.51	0	3.89
[^57^Fe], mg	2.36	0	0	2.36	0	0
Added Fe^4^						
Unlabeled Fe, mg	0	6.87	0	0	6.87	0
[^54^Fe], mg	0	0	5.98	0	0	5.98
[^58^Fe], mg	0	3	0	0	3	0
Total Fe, mg (planned)^3^	9.87	9.87	9.87	9.87	9.87	9.87
Total Fe, mg (analyzed)^5^	14.6	13.2	14.8	9.87	12.6	13.1
Phytate:Fe molar ratio^5^	0.41	0.12	0.28	3.12	2.22	2.31

1 Porridge made of refined maize flour—noninhibitory food matrix.

2 Porridge made of nonrefined maize flour—inhibitory food matrix.

3 Fe content based on chemical analysis before study.

4 Added as FeSO_4_.

5 Fe content based on posterior analysis of phytate and Fe in study meals.

#### Extrinsic labeling of experimental meals

Isotopically enriched elemental iron (^54^Fe, 99.7% enrichment; and ^58^Fe, 99.9% enrichment) was purchased from Chemgas. The ^54^Fe-metal and ^58^Fe-metal powders were used to produce labeled ^54^FeSO_4_ and ^58^FeSO_4_ by dissolution in 0.1 M sulfuric acid to concentrations of 0.111 M and 0.052 M, respectively. The solutions were flushed with argon to keep the iron in the Fe^2+^ state. Shortly before study meal administration, gravimetrically measured tracer doses of the labeled FeSO_4_ solutions were mixed into the study meals.

#### Preparation of experimental meals with crickets

Frozen crickets were blanched for 2 min, chilled, and then quickly frozen to −40°C in a shock freezer. After that, the product was loaded into the freeze drier (Lyofast S08 and Super Modulyo, Edwards) set to −20°C, a vacuum was applied (±1–50 mbar absolute), and the temperature was slowly increased to +27°C. The condenser temperature was −60°C. The drying process lasted for ∼7 d. The weight of freeze-dried crickets was 24% of the initial fresh weight. The freeze-dried crickets were packaged in vacuum-sealed plastic bags and stored at room temperature. A few days before administering study meals, freeze-dried crickets were ground into a coarse cricket powder with a kitchen grinder. The cricket powder was stored in the freezer (−18°C) until meal preparation and administration.

#### Biochemical analyses

Hemoglobin concentration was assessed in EDTA whole blood by the ADVIA 2120i hematology analyzer (Siemens Healthineers). A latex-enhanced immunoturbidimetric assay was used to assess serum CRP concentration (Atellica, Siemens Healthineers). The serum concentrations of ferritin and sTfR were measured by ELISA using the Vista Dimension 1500 (Siemens Healthineers). Normal ranges of blood concentrations were specified by common clinical standards or assay specifications as follows: hemoglobin, 12–16 g/L; serum ferritin, 15–200 µg/L; sTfR, 2.9–8.3 mg/L; CRP <5 mg/L. Whole blood samples collected on crossover and endpoint visits were mineralized by microwave-assisted digestion (TurboWave, MLS) in HNO_3_, followed by separation of the iron from the blood matrix by anion-exchange chromatography and a subsequent precipitation step with ammonium hydroxide. All isotopic analyses were performed by multicollector inductively coupled plasma (ICP)-MS (Neptune, Thermo Fisher Scientific) at the Laboratory for Human Nutrition, ETH Zürich, Switzerland (20). Iron content and isotopic enrichment of cricket feed, crickets, and duplicate meals were analyzed likewise. The phytate content of the duplicate meals was assessed by the modified Makower method (21).

### Calculations and statistical analyses

Fractional iron absorption was calculated based on the shift in iron isotope ratios and the estimated amount of circulating iron using isotope dilution principles (21), assuming that 80% of the absorbed label would be incorporated into the erythrocytes 14 d after meal intake (22). Circulating iron was calculated based on each participant's hemoglobin concentration and estimated blood volume (23). Total iron absorption was calculated by multiplying each individual's meal-specific fractional iron absorption with the measured iron content of each meal. Data analysis was performed in MS Excel 2016 (Microsoft) and SPSS version 25 (IBM Corporation), whereas the graphical presentation of data was produced with GraphPad Prism version 5.04 (GraphPad Software Inc.).

All variables and model residuals were checked for normality by visual inspection of their distributions and Q-Q plots. Pearson correlation coefficients were calculated to explore the relation between iron status (average concentration of serum ferritin and sTfR) and fractional iron absorption from the study meals. Linear mixed models with an unstructured covariance matrix were used to explore changes in iron status indicators over time, as well as to assess differences in fractional and total iron absorption between meals, with time point and meal order as fixed effects. The effect of covariates on the model fit was explored by adding these as fixed effects (e.g., flour type) or random effects (e.g., iron status indicators, inflammation markers) to the model. The model with the lowest Akaike Information Criterion (AIC) was finally selected as the best model fit. Mean differences in fractional iron absorption with 95% CIs generated by the linear mixed model were used to interpret the results. No corrections for multiple comparisons were made because of the nonconfirmatory nature of the study.

### In vitro study

In addition to the human in vivo study, we also conducted in vitro studies to evaluate potential inhibitors of mineral absorption present in crickets, i.e., chitin, its digestive breakdown product chitosan, and calcium. Chitin constitutes a large part of the exoskeleton of crickets, which may inhibit iron absorption in addition to phytate, calcium, and polyphenols found in insects (24). In vitro digestion was performed to assess the bioaccessibility of iron according to the INFOGEST protocol (24), with some modifications. All samples were prepared in triplicate. The enzyme salivary amylase was excluded in the oral phase because the samples contained no or only little starch. Each sample (1.25 g) was mixed with 1.25 mL MilliQ water to make a paste-like consistency, followed by adding 2 mL simulated salivary fluid (SSF), 12.5 µL CaCl_2_, and 462.5 µL MilliQ. The mixture was incubated for 2 min at 37°C. The gastric and intestinal phases were performed as described in the protocol. Porcine pepsin, pancreatin, and bile extract were purchased from Sigma Aldrich. A blank sample was prepared for each run, consisting of 1.25 mL MilliQ water only. At the end of the simulated intestinal phase, the mixture was cooled in an ice bath for 15 min, followed by centrifugation at 5000 × *g*for 15 min at room temperature. The supernatant was then stored at −21°C until further analysis for soluble iron.

A range of samples were prepared to investigate the inhibitory effect of chitin, chitosan, and calcium on iron absorption. Chemicals, i.e., FeSO_4_ [Chemical Abstract Service (CAS): 7782-63-0] and CaSO_4_ (CAS: 10101-41-4), both with ≥99% purity; and chitin (CAS: 1398-61-4) and chitosan (CAS: 9012-76-4) (both from shrimp shells), were all purchased from Sigma-Aldrich. Chitin from shrimp has a fairly similar structure to chitin present in crickets (25). Because the sample weight was always 1.25 g, the amount of iron (as FeSO_4_) added to a sample was limited by the weight of other components in the sample. We took the mass ratio of compounds as present in 100 g dry matter of crickets (iron, 6 mg; chitin, 7.5 g; calcium, 115 mg) as guidance for deciding on the amounts of antinutrients and iron to be added to each sample (**Table 2**). Crickets used for the in vitro study were taken from the same batch as for the human study.

**TABLE 2:** Composition of digestive samples and mean (95% CI) in vitro bioaccessibility of iron^1^

Sample	Cricket powder, mg	Chitin, mg	Chitosan, mg	CaSO_4_,^2^ mg	Ca,^3^ mg	FeSO_4_,^4^ mg	Fe, mg	Iron bioaccessibility,^5^ % (95% CI)
Blank	—	—	—	—	—	—	—	—
1	—	—	—	—	—	1250	250	71.1 (63.7, 78.4)
2	1250	—	—	—	1.44	—	0.075	87.5 (85.5, 89.5)
3	750	—	—	—	0.86	500	100	14.2 (5.41, 22.9)
4	—	988	—	—	—	11.8	2.37	0.42 (—)
5	—	997	—	—	—	2.99	0.60	−0.92 (−1.56, −0.28)
6	—	—	997	—	—	2.99	0.60	−2.00 (−2.03, −1.98)
7	—	—	—	1112	259	138	27.8	0.71 (0.21, 1.22)
8	—	—	—	1200	279	49.8	10.0	1.38 (1.17, 1.60)
9	—	1007	—	229	53.3	14.3	2.86	0.13 (−0.61, 0.87)
10	—	1157	—	87.6	20.4	5.46	1.10	−0.78 (−0.93, −0.62)
11	—	1199	—	45.4	10.6	5.66	1.14	−0.51 (−1.44, 0.42)

1 *n* = 3.

2 As calcium sulfate dihydrate (23% calcium).

3 In addition, 0.421 mg CaCl_2_(H_2_O)_2_ (= 0.11 mg Ca) was added per INFOGEST protocol.

4 As ferrous sulfate heptahydrate (20% iron).

5 Adjusted for blank.

Iron content of input material and supernatants for the samples containing FeSO_4_ (positive control) and ground crickets + FeSO_4_ were analyzed by the Soil Chemistry Laboratory at Wageningen University, Netherlands. Samples were destructed using HNO_3_–HCl (aqua regia), after which 15 mL of supernatant was pipetted in a digestion tube. After adding nitric acid and hydrochloric acid, the sample was heated in a microwave [MarsXpress, CEM corporation (Beun de Ronde)]. After settling the undissolved particles, the supernatant was analyzed for iron using ICP–optical emission spectrometry (OES) (Thermo iCAP 6500 DV, Thermo Fisher Scientific). All other in vitro digest samples were analyzed at the Laboratory for Human Nutrition, ETH Zürich, Switzerland, by ICP-MS (iCap RQ, Thermo Fisher Scientific) after appropriate dilution with 1% nitric acid.

Mineral bioaccessibility, defined as the soluble mineral fraction in the supernatant, was calculated using the following formula:

(1) $$\begin{eqnarray*}
&&{\rm \% \,\,Fe\,\,bioaccessibility}\\
&& = \,\,\frac{{{\rm{Fe\,\,in\,\,digested\,\,sample\,\,}}\left( {{\rm{mg}}} \right){\rm{\,\,}} - {\rm{\,\,Fe\,\,in\,\,blank\,\,sample}}\left( {{\rm{mg}}} \right)}}{{{\rm{Fe\,\,in\,\,the\,\,undigested\,\,sample\,\,}}\left( {{\rm{mg}}} \right)}}\\
&& *100
\end{eqnarray*}$$

The average iron content of the blank was subtracted from all sample results included in the same run of 6 samples. Replicates of each condition (*n* = 3) were used to calculate mean bioaccessibility with 95% CIs.

## Results

**FIGURE 1:**
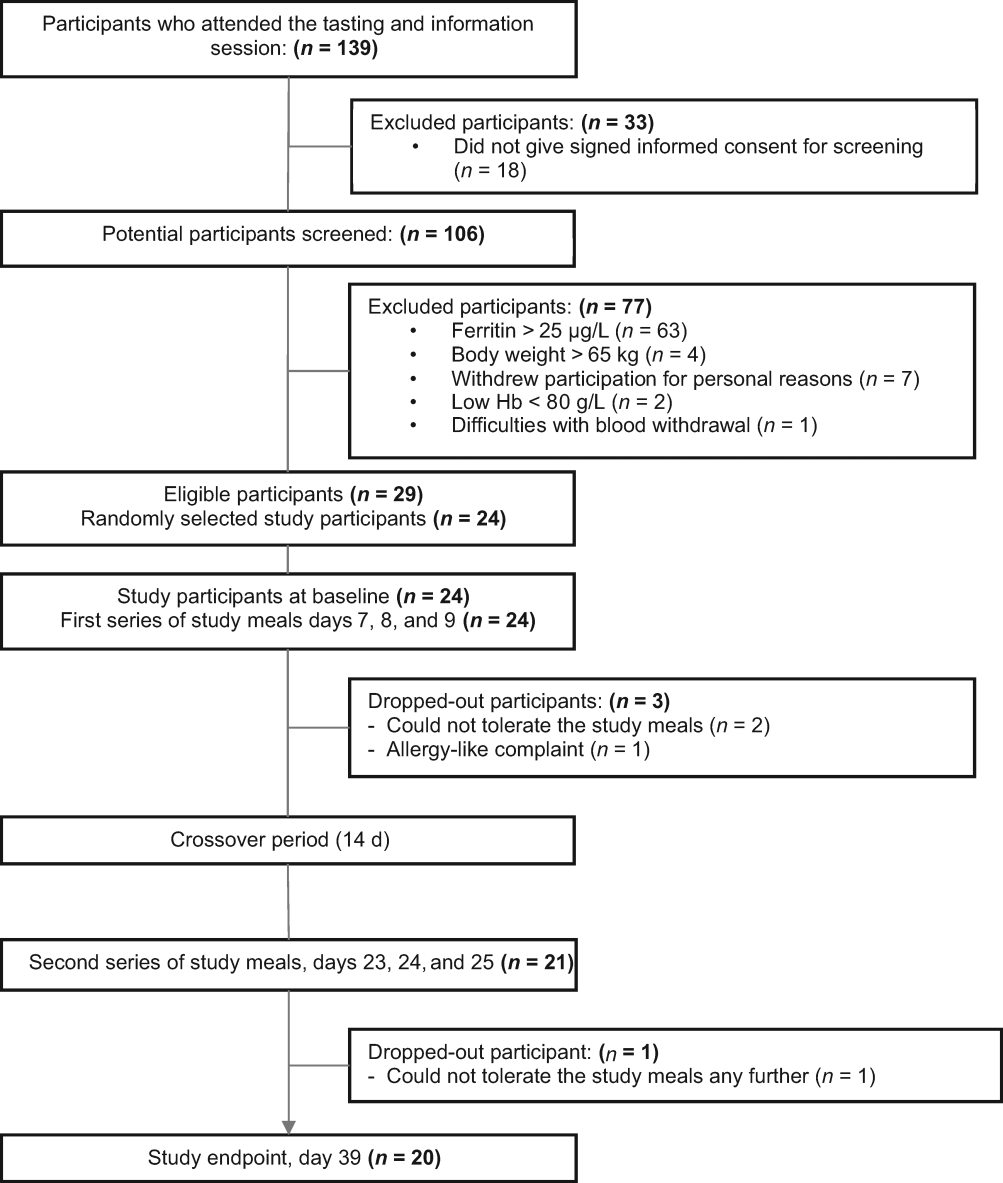
Flowchart of participant enrolment and dropout. Hb, hemoglobin.

As shown in **Figure 1**, 139 females attended 1 of the information sessions, out of whom 106 were screened for eligibility. Prospective participants were excluded (*n* = 77) mostly because of having a serum ferritin concentration >25 µg/L (*n* = 63), whereas 2 participants were excluded because they were referred for treatment by the study physician owing to their low hemoglobin concentration (<80 g/L). Out of the 29 iron-depleted eligible participants, 24 were randomly selected and enrolled in the study. A total of 4 participants dropped out during the study, either related to allergy complaints (*n* = 1) or because they could not tolerate the relatively large portion sizes of the experimental meals (*n* = 3). The remaining participants (*n* = 20) had normal mean ± SD BMI (21.6 ± 1.90 kg/m^2^) and hemoglobin concentration (132 ± 11.3g/L) at the start of the study (**Table 3**). None of the participants had severe anemia, but the hemoglobin concentration of 2 participants was <120 g/L. Serum ferritin concentration was marginal, as planned, and 8 participants (40%) had a serum ferritin concentration <15 µg/L at the onset of the study. None of the participants showed signs of acute inflammation at any time point.

**TABLE 3:** Characteristics of participants who completed the study^1^

Variable	Baseline	Day 23	*P* value^2^	Day 39	*P* value^2^
Age, y	24.7 ± 2.9				
Body weight, kg	57.3 ± 5.8	57.5 ± 5.9			
Body height, cm	163 ± 7.5				
BMI, kg/m^2^	21.6 ± 1.9	21.7 ± 1.9			
Hemoglobin, g/L	132± 11.3	131± 11.3	0.424	132± 11.3	0.625
Serum ferritin, µg/L	15.4 ± 6.5	18.9 ± 10.6	0.063	19.0 ± 2.9	0.099
Soluble transferrin receptor, mg/L	5.0 ± 1.4	6.1 ± 1.9	<0.001	5.9 ± 1.8	0.002

1 *n* = 20. Values are means ± SDs.

2 *P* value for difference from baseline, by linear mixed models with an unstructured covariance matrix and with time point as a fixed effect.

**FIGURE 2:**
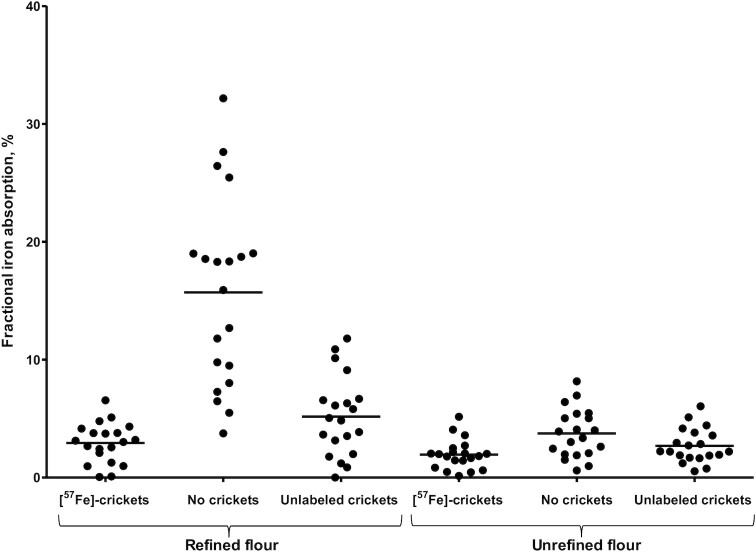
Crude fractional iron absorption from each study meal, with dots representing individual values and horizontal lines representing means (*n* = 20). Meals were prepared from refined or nonrefined maize flour, with either intrinsically ^57^Fe-labeled house crickets, no crickets plus ^58^FeSO_4_, or unlabeled house crickets plus ^54^FeSO_4_.

**Figure 2** displays crude fractional iron absorption from the study meals for each of the 20 participants. Despite randomization of all meal orders across the 2 time series, mean fractional iron absorption was lower in the second series of meals (3.82%; 95% CI: 2.96%, 4.68%) than in the first series of meals (6.99%; 95% CI: 5.21%, 8.78%; *P* = 0.003). When seeking possible explanations, we found that mean serum ferritin concentration had increased from 15.4 ± 6.5 µg/L at baseline to 18.9 ± 10.6 µg/L at the start of the second series of meals (Table 3), which would be indicative of a small increase in iron status. Serum ferritin concentration correlated negatively with fractional iron absorption (**Table 4**). In contrast, serum sTfR concentration had also increased from baseline to the start of the second series of meals (Table 3), which would be indicative of a decrease in iron status, but correlation coefficients with fractional iron absorption were low (Table 4). In linear mixed models, the best model fit was achieved by only adding time and time × meal as fixed covariates, without including any indicators of iron status.

**TABLE 4:** Pearson correlation coefficients of iron status indicators with fractional iron absorption from study meals^1^

	Meals with refined maize flour^2^			Meals with nonrefined maize flour^3^		
	[^57^Fe]-crickets	No crickets	Unlabeled crickets	[^57^Fe]-crickets	No crickets	Unlabeled crickets
Serum ferritin, µg/L						
Correlation coefficient	−0.517	−0.289	−0.559	−0.383	−0.368	−0.173
*P* value	0.020	0.216	0.010	0.095	0.111	0.467
Soluble transferrin receptor, mg/L						
Correlation coefficient	0.121	0.261	0.368	0.200	0.253	0.189
*P* value	0.610	0.267	0.111	0.398	0.282	0.425

1 *n* = 20.

2 Porridge made of refined maize (corn) flour—noninhibitory food matrix.

3 Porridge made of nonrefined maize (corn) flour—inhibitory food matrix.

Estimated marginal mean fractional iron absorption from the refined flour meal without crickets was distinctly higher (14.2%; 95% CI: 12.9%, 15.5%) than fractional iron absorption from any of the other meals, which ranged from 2.2% to 4.9% (**Table 5**). Mean fractional iron absorption from ^57^Fe-labeled house crickets with refined maize porridge (3.06%; 95% CI: 1.75%, 4.37%) did not substantially differ from that from ^57^Fe-labeled house crickets with unrefined maize porridge (2.17%; 95% CI: 0.57%, 3.76%). Iron absorption from the meal with unlabeled crickets added to refined maize flour was higher than from the meal with ^57^Fe-labeled crickets (mean difference: 1.86%; 95% CI: 0.43%, 3.29%). None of the unrefined maize meal porridges showed any differences in iron absorption.

**TABLE 5:** Estimated marginal means and mean differences (95% CIs) of fractional and total iron absorption from study meals^1^

	Fractional iron absorption, %		Total iron absorption,^2^ mg	
Meal type	Mean (95% CI)	Mean difference (95% CI)	Mean (95% CI)	Mean difference (95% CI)
Refined maize flour				
[^57^Fe]-crickets	3.06 (1.75, 4.37)	−11.1 (−12.6, −9.68)	0.43 (0.25, 0.60)	−1.46 (−1.65, −1.27)
No crickets	14.2 (12.9, 15.5)	Reference	1.88 (1.71, 2.06)	Reference
Unlabeled crickets	4.92 (3.59, 6.25)	−9.29 (−10.8, −7.77)	0.72 (0.55, 0.90)	−1.16 (−1.36, −0.96)
Nonrefined maize flour				
[^57^Fe]-crickets	2.17 (0.57, 3.76)	−0.56 (−2.44, 1.31)	0.22 (0.01, 0.43)	−0.07 (−0.32, 0.18)
No crickets	2.73 (1.18, 4.28)	Reference	0.29 (0.09, 0.50)	Reference
Unlabeled crickets	2.61 (1.09, 4.14)	−0.12 (−2.03, 1.78)	0.34 (0.14, 0.55)	0.05 (−0.21, 0.31)

1 *n* = 20. Estimated marginal means and mean differences from pairwise comparisons generated by linear mixed models with time point and meal order as fixed effects and with time and time × meal added as fixed covariates to the model.

2 Calculated by multiplying the mean fractional iron absorption with the iron content of each meal, as analyzed (Table 1).

### In vitro study

**FIGURE 3:**
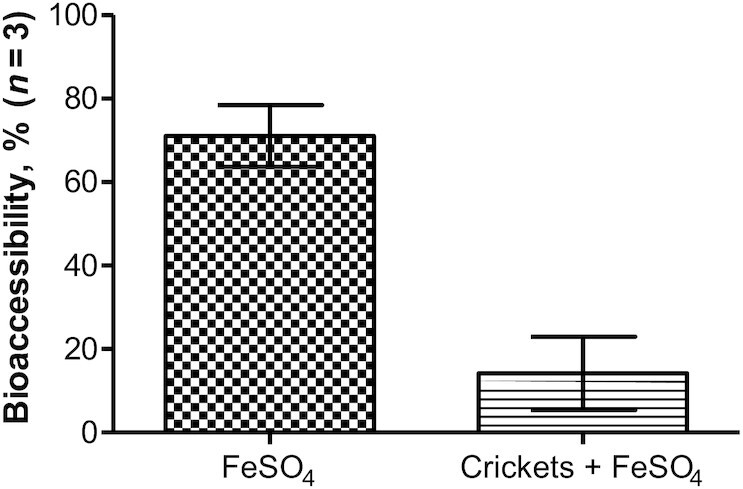
Mean in vitro bioaccessibility of FeSO_4_ with and without crickets added to the digest (*n* = 3). Error bars represent 95% CIs.

A total of 71% (95% CI: 64%, 78%) of iron was bioaccessible when only ferrous sulfate was added to the digest (positive control), whereas this decreased to 14% (95% CI: 5.4%, 23%) when adding cricket powder to the ferrous sulfate (**Figure 3**). Cricket powder alone showed a bioaccessibility of 88% (95% CI: 86%, 90%), but it should be noted that the small amount of iron in the sample (60 ppm) was below the limit of quantification (Table 2). With either chitin, chitosan, calcium alone, or a combination of chitin and calcium added to the digests in various mass ratios to iron (based on the ratio in which they appear in crickets), the bioaccessibility of iron was close to 0 with negligible differences between conditions (Table 2).

## Discussion

We found that the fractional absorption of iron from intrinsically labeled house crickets added to maize porridge was generally low. This was unexpected and most apparent when the crickets were consumed as part of a noninhibitory meal compared with the corresponding reference meal with added ferrous sulfate. Moreover, we did not find evidence that the absorption of iron added extrinsically to the meals was improved when adding (unlabeled) crickets. This suggests that iron from crickets is absorbed in a largely similar fashion to nonheme iron from other food sources.

In our in vitro digestion studies, we found that both chitin and its breakdown product chitosan inhibited the bioaccessibility of iron. Chitin is an N-acetyl-β-d-glucosamine polymer that is one of the primary components of insect exoskeletons and crustacean shells (26), and the cuticle of house crickets is rich in chitin (25, 27). However, proper comparison of the amount of chitin between species is hampered by lack of agreement between analytical methods (28). Human gastric juice has been shown to contain acidic mammalian chitinase capable of degrading chitin, but it is unknown to what extent this is effective in vivo (28, 29). Purified chitin and its deacetylation product chitosan are increasingly used as food additives and dietary supplements, for example for chelation of fat, thereby making use of chitin's indigestibility (26, 30–32). A human intervention study showed that consumption of cricket powder for 2 wk increased the probiotic gut bacterium *Bifidobacterium animalis* 5.7-fold and decreased systemic inflammation, which was attributed to the presence of chitin and other indigestible fibers (33).

To our knowledge, neither chitin nor chitosan have been reported as inhibitors of iron absorption in humans before. Both chitin and chitosan are insoluble chelators that can bind lipids, cholesterol, and fat-soluble vitamins. Studies in rats have shown that chitosan interferes with calcium metabolism, although zinc absorption appeared to be unaltered (30). Other studies showed that prolonged dietary chitin or chitosan supplementation resulted in a negative iron balance and low hemoglobin concentration in rats (34, 35). The effect of purified chitin and chitosan on iron absorption in humans remains to be assessed. If confirmed, the inhibitory effect of chitin could best be avoided by separating the crickets into protein-, lipid-, and chitin-enriched fractions for further use. Procedures to do so have been developed (36), and protein-enriched products without chitin are already on the market for lesser mealworm (*Alphitobius diaperinus*).

As expected, study meals made of nonrefined maize flour showed a lower iron absorption than meals made of refined maize flour, attributable to their higher phytic acid content (4). Phytic acid, or inositol polyphosphate, is well-known to chelate divalent metals such as iron to form nonsoluble complexes which are poorly digested in the duodenum. Because phytates could have been present in the cricket gut owing to the cereal-based feed on which they were reared, this feed was removed for 24 h before harvesting. The phytate:iron molar ratios of the refined study meals were somewhat higher when insects were added (Table 1), but ratios were below the threshold of 1 above which iron absorption is known to decline rapidly (2). Phytate:iron molar ratios of all the nonrefined meals were well above 1, which was reflected in the low absorption values. Hence, phytate may have masked any inhibition of iron absorption by chitin in the nonrefined meals.

Like phytates, various bioactive substances can also form insoluble and nondigestible complexes with divalent cations (37, 38). Crickets contain a range of bioactive substances, such as polyphenols, tannins, flavonoids, saponins, alkaloids, and oxalates (39–41), which can all potentially limit mineral bioavailability. The house crickets used in this study also contained a relatively high amount of calcium due to the enriched chicken feed on which they were reared. In short-term dietary studies, the addition of 150 mg Ca to meals led to a 50% reduction of iron absorption (37, 42), but this has not been confirmed in longer-term studies and may be highly dependent on other components in a meal (42). The cricket meals in our study contained an analyzed amount of 100–170 mg Ca compared with 25 mg for the noncricket meals; hence, this also may have reduced iron absorption, in line with the in vitro studies. Besides this, we found a difference in iron absorption between the meals prepared from refined flour with 100 g of ^57^Fe-labeled crickets added (mean: 3.06%) and with 50 g of unlabeled crickets added (mean: 4.92%) (Table 5), which may be due to only half the amount of inhibitors being contributed by the crickets in the latter meal.

Because dietary protein from animal sources is known to enhance nonheme iron absorption (43), we explored the effect of adding crickets to a meal on the absorption of extrinsically added iron. The enhancing effect of protein on iron absorption is attributed to sulfur-containing amino acids that keep iron in solution and available for absorption in the digestive tract, but may also be explained by a reduction of the inhibitory effects of antinutrients or by stimulating gastric acid production (37, 43). Contrary to our prior expectation, we found that less iron was absorbed from refined maize porridge when crickets were added. Previous studies have shown that protein from sources other than meat, such as dairy, eggs, and soybean, also inhibited iron uptake (43). These contradictions may be due to differences in amino acid or peptide composition. Even though house crickets’ protein content and amino acid composition are largely comparable with those of meat (44), addition of crickets to the iron-enriched maize porridges did not enhance but rather reduced iron absorption.

To our knowledge, ours is the first in vivo study that assessed the bioavailability of iron from edible insects in humans. We earlier reported our findings on the same study as a conference abstract, in which we reported crude estimates of iron absorption based on all participants with data points (*n* = 24), which therefore deviate slightly from values presented in this article (45). We used a rigorous crossover study design, thereby minimizing between-subject effects in the study outcomes. We also applied an isotope dilution method with crickets that had been intrinsically labeled with iron. This approach allowed the label to be built into the body structures of the crickets, taking any effects of the food matrix on iron absorption into account. The experimental meals differed slightly in size and macronutrient content, and meal analysis also showed small differences in iron content between meals. Nevertheless, conclusions based on the total amount of absorbed iron from the experimental meals (Table 5) would not differ from results based on fractional iron absorption. The incidental use of artificial sweeteners in the meals by some of the participants may have altered iron absorption, for which we did not account. The difference in iron absorption between the first and second series of meals could not be explained by changes in iron status over time, and remains unexplained. Although the total iron absorbed from the meals provided 15%–25% of the iron requirements of participants, it should be noted that the meals were of an experimental nature and are unlikely to be consumed in similar quantities in real life.

An important question remains: can our findings be extrapolated to other edible insect species given the large differences in insect body composition? A previous in vitro study noted that, despite the high content and solubility of iron in crickets, cellular uptake was lower than for grasshoppers, mealworms, and buffalo worms for unexplained reasons (19). Although our in vitro study showed very high bioaccessibility of iron from cricket powder (88%), this should be interpreted with caution because the amount of iron present in the digests was very low and close to the analytical limit of detection.

In conclusion, our study indicates that the absorption of iron from house crickets by humans is modest. This can be explained by the presence of chitin, although other antinutrients may also play a role. Our study was of an exploratory nature and therefore needs further confirmation by future studies. Because the composition of insects varies widely, our results may not be indicative for iron absorption from edible insects other than crickets that contain lower amounts of chitin and other antinutritional factors. Given the increased use of chitin and chitosan in the human food chain, their effect on mineral absorption and metabolism remains to be further explored.

## Data Availability

Data described in the article, code book, and analytic code will be made publicly and freely available without restriction at https://data.4tu.nl/, doi:10.4121/19111334.

## Notes

Author disclosures: The authors report no conflicts of interest

Supported by Wellcome Trust grant 106856/Z/15/Z (to MD, JJAvL, AM-B, and MBZ). The Wellcome Trust had no role in the design or analysis of the study, nor in the writing of this article.

Abbreviations used: CAS, Chemical Abstracts Service; CRP, C-reactive protein; ICP, inductively coupled plasma; sTfR, soluble transferrin receptor.
